# Extracellular Vesicles: New Classification and Tumor Immunosuppression

**DOI:** 10.3390/biology12010110

**Published:** 2023-01-10

**Authors:** Mona Sheta, Eman A. Taha, Yanyin Lu, Takanori Eguchi

**Affiliations:** 1 Department of Dental Pharmacology, Graduate School of Medicine, Dentistry and Pharmaceutical Sciences, Okayama University, Okayama 700-8525, Japan; 2 Department of Cancer Biology, National Cancer Institute, Cairo University, Cairo 11796, Egypt; 3 Department of Biochemistry, Faculty of Science, Ain Shams University, Cairo 11566, Egypt; 4 Department of Oral and Maxillofacial Surgery, Stomatological Center, Peking University Shenzhen Hospital, Shenzhen 518036, China

**Keywords:** extracellular vesicle, exosome, autophagy, amphisome, matrix vesicle, cellular communication, tumor microenvironment, immunosuppression, immune evasion, therapy resistance

## Abstract

**Simple Summary:**

Extracellular vesicles (EVs) are cell-derived membrane-surrounded vesicles that carry bioactive molecules and deliver them to recipient cells. Classical EVs are exosomes, microvesicles, and apoptotic bodies. This review classifies classical and additional EV types, including autophagic EVs, matrix vesicles, and stressed EVs. Of note, matrix vesicles are key components interacting with extracellular matrices (ECM) in the tumor microenvironment. We also review how EVs are involved in the communication between cancer cells and tumor-associated cells (TAC), leading to establishing immunosuppressive and chemoresistant microenvironments. These include cancer-associated fibroblasts (CAF), mesenchymal stem cells (MSC), blood endothelial cells (BEC), lymph endothelial cells (LEC), and immune cells, such as tumor-associated macrophages (TAM), tumor-associated neutrophils (TAN), dendritic cells, natural killer cells, killer T cells, and immunosuppressive cells, such as regulatory T cells and myeloid-derived suppressor cells (MDSC). Exosomal long noncoding RNA (lncRNA), microRNA, circular RNA, piRNA, mRNA, and proteins are crucial in communication between cancer cells and TACs for establishing cold tumors.

**Abstract:**

Extracellular vesicles (EVs) are cell-derived membrane-surrounded vesicles carrying various types of molecules. These EV cargoes are often used as pathophysiological biomarkers and delivered to recipient cells whose fates are often altered in local and distant tissues. Classical EVs are exosomes, microvesicles, and apoptotic bodies, while recent studies discovered autophagic EVs, stressed EVs, and matrix vesicles. Here, we classify classical and new EVs and non-EV nanoparticles. We also review EVs-mediated intercellular communication between cancer cells and various types of tumor-associated cells, such as cancer-associated fibroblasts, adipocytes, blood vessels, lymphatic vessels, and immune cells. Of note, cancer EVs play crucial roles in immunosuppression, immune evasion, and immunotherapy resistance. Thus, cancer EVs change hot tumors into cold ones. Moreover, cancer EVs affect nonimmune cells to promote cellular transformation, including epithelial-to-mesenchymal transition (EMT), chemoresistance, tumor matrix production, destruction of biological barriers, angiogenesis, lymphangiogenesis, and metastatic niche formation.

## 1. Introduction

Extracellular vesicles (EVs) are cell-derived membrane-surrounded vesicles carrying various types of molecules. These EV cargoes are often pathophysiological biomarkers and alter recipient cell fates in local and distant tissues [1]. Classical EVs are exosomes, microvesicles, and apoptotic bodies, while recent studies discovered autophagic EVs, stressed EVs, and matrix vesicles [2,3]. This review classifies classical and new EVs and non-EV nanoparticles (Section 2.1). Of note, kinases (ULK1, VPS34, SRC) [4,5] and a kinase-specific chaperone CDC37 play key roles in exosome biogenesis and secretion [3,6] (Section 2.2).

**Figure 1:**
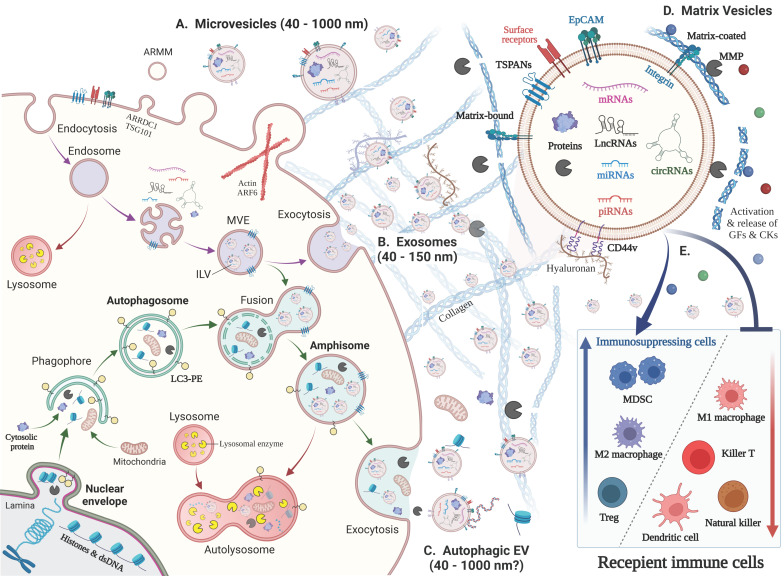
Biogenesis of various EV types and their immunoregulatory roles. (Left) Biogenesis of various EV types. (**A**) Microvesicles (40–1000 nm) arise from the outward budding and shedding of the plasma membrane. Small microvesicles called ARMM were recently discovered. (**B**) Exosomes (40–150 nm) are secreted via exocytosis of intraluminal vesicles (ILVs) by plasma membrane fusion with multivesicular endosomes (MVE) with the plasma membrane (purple arrow). (**C**) Autophagosome fusion with MVEs generates ‘amphisomes’, whose fusion with the plasma membrane secretes autophagic EVs and vesicle-free cytosolic and nuclear molecules, such as histones and dsDNA (green arrows). This pathway is called ‘exophagy’, a hybrid of exosomes and autophagy. Mitochondrial autophagy is called ‘mitophagy,’ which may result in the secretion of mitochondria. (Upper right) EVs contain a lipid bilayer membrane that protects encapsulated materials, such as proteins, nucleic acids, lipids, and metabolites. The EV surface contains membrane-bound and transmembrane proteins, such as tetraspanins (TSPANs: CD63, CD9, CD81, etc.) and integrins, which bind to matrix proteins. (**D**) ‘Matrix vesicle’ is a general term for matrix-bound vesicles and matrix-coated vesicles. MMPs cleave matrix components, such as collagen, to release EVs, growth factors (GFs), and chemokines (CKs). (**E**) Immunoregulatory roles of cancer cell-derived EVs. (i) The suppression and apoptosis of killer T cells, natural killer cells, and dendritic cells; (ii) activation of immunosuppressing cells, such as MDSCs and Tregs; and (iii) polarization of macrophages from the M1 to M2 type.

Autophagy is largely involved in EV biogenesis and release (Section 2.3). Autophagosome can fuse with endosomes to generate amphisomes, which is then secreted as autophagic EVs [2] (Figure 1). We also mention the involvement of epithelial-to-mesenchymal transition (EMT) and cancer stemness in EV biogenesis and release (Section 2.4). Matrix vesicles and extracellular matrix (ECM) are key components interacting with each other in the tumor microenvironment (TME) (Section 2.5). Matrix moonlighting metalloproteinases (MMPs) are involved in the generation and function of matrix vesicles [7,8].

Molecular transfer activity to recipient cells and molecular delivery to target tissues are key functions of EVs. Protein S-palmitoylation enables the proteins to associate with lipid membranes in cells and EVs, thus contributing to sorting the proteins to EVs and delivering them to recipient cells [9] (Section 2.6). Many protein markers (such as CD9, CD63, and CD81) and stem cell markers (such as EpCAM, CD44 variants, and CD133) have been established for defining EVs [10]. Nevertheless, numerous new biomarkers of diseases and pathophysiological conditions are currently being developed from EVs. Noncoding RNAs, including long noncoding RNA (lncRNA), microRNA (miRNA), circulating RNA (circRNA), and PIWI-interacting RNA (piRNA), are found as biomarkers and functional RNA (Section 2.7).

The cell-autonomous growth of cancer cells is one of the main causes of tumor development. In addition, tumor growth, progression, and resistance largely depend on their microenvironment status. Tumors are surrounded by various cells, such as cancer-associated fibroblasts (CAF), adipocytes, blood vessels, lymphatic vessels, and immune cells. EVs mediate the intercellular communication between cancer cells and various types of tumor-associated cells (TAC) in the tumor microenvironment (TME) (Section 3).

Cancer cell-derived EVs: (i) promote tumor angiogenesis, extravasation, and intravasation by regulating blood endothelial cells (BEC) and lymphatic endothelial cells (LEC); (ii) promote the differentiation of functions of immunosuppressive cells, such as myeloid-derived suppressor cells (MDSC) and regulatory T (Treg) cells; (iii) change the differentiation or polarity of various cell types into pro-tumorigenic, immunosuppressive, anti-inflammatory, and chemoresistant phenotypes. These include the differentiation/polarization of fibroblasts into CAF, monocytes, and tumor-associated macrophages (TAM) into M2-polarized TAM and neutrophils into N2-polarized neutrophils; (iv) induce apoptosis in dendritic cells (DC) and killer T cells; and (v) disable natural killer (NK) cells. Thus, tumor EVs are essential for establishing “cold” tumors that are not or less responsive to immunotherapy (Section 4). Furthermore, tumor microenvironmental cells, such as CAF, MSC, TAM, and N2 cells, produce EVs to deliver bioactive molecules to cancer cells for inducing chemoresistance, immunotherapy resistance, dormancy, stemness, and EMT. Thus, these TACs and cancer cells mutually communicate via EVs to establish an immunosuppressive and resistant microenvironment.

## 2. New Classification, Biogenesis and Functions of EVs

### 2.1. New Classification and Terminology of EVs

EVs are classified based on their biogenesis mechanism (e.g., exosome, microvesicle, apoptosome, and autophagic EV) [2]; concept (e.g., oncosome, matrix vesicle, stress EV, and migrasome) [3,7,11,12,13]; and size (e.g., small EV and large EV) [1] (Table 1). Exosomes are endosome-originated EVs generated through three steps: biogenesis, transport, and release [14] (Figure 1). Microvesicles are formed through direct outward budding and shedding from the plasma membrane [15,16]. Apoptotic bodies are generated in the process of apoptosis. These three classical EV types are mutually exclusive based on their biogenesis mechanism. However, additional EVs are more conceptual and not entirely mutually exclusive. Moreover, advanced techniques are needed to separate exosomes, microvesicles, and other EV types.

The term ‘exosome’ is so designated as it originated from combining the terms endosomes and secreted via exocytosis. It is experimentally challenging to distinguish exosomes from other EV types, such as microvesicles (100–500 nm) released by plasma membrane shedding. However, the term ‘exosome’ has been used frequently in EV research without confirming exocytosis. For this reason, most experts recently have recommended calling exosomes small EVs (sEV), which is clearly stated in the position paper [1]. Additionally, recent studies discovered small and large exosomes termed Exo-S (40–80 nm) and Exo-L (80–150 nm) [1,17] Exo-L contained CD9, while Exo-S contained CD63 secreted by macrophage-like cells differentiated from THP-1 cell line [18].

Recently, a reassessment of exosome composition revealed that (i) cytosolic DNA and nucleosomes (dsDNA/histones) are secreted with exosomes via the autophagy-amphisome pathway. Nucleosomes are released via active release of amphisome (LC3+) and passive release by cell death. Histones are also detected as non-vesicular (NV) proteins. (ii) Small microvesicles (~40–100 nm) released from cells with ARRDC1 (arrestin-domain-containing protein 1) and TSG101 are designated ARMM (ARRDC1-mediated microvesicles), distinguished from other microvesicle types: classical large-microvesicles (~150–1000 nm) and large oncosomes (1–10 µm), both of which contain Annexin A1. (iii) Human cells release Argonaute 1–4 and major vault protein (MVP) independently of exosomes [2] (Table 1).

Exomeres are non-EV nanoparticles. Exomeres contain beta-galactoside a2, 6-sialyltransferase 1 (ST6Gal-I), and amphiregulin (AREG) [19]. ST6Gal-I is transported from exomeres to recipient cells, conferring a cancer stem cell phenotype [20,21].

### 2.2. Exosome

#### 2.2.1. Multivesicular Endosome (MVE) Biogenesis and Exosome Secretion

For the biogenesis of multivesicular endosomes (MVE), also known as multivesicular bodies (MVBs) containing future exosomes, proteins are transported from the trans-Golgi network (TGN) (e.g., MHC class II molecules) or internalized from the cellar surface (e.g., activated growth factor receptors) [22] (Figure 1). These proteins are ubiquitylated at their cytosolic domains; however, not all proteins require ubiquitinylation to be fused with early exosomes. During the maturation process that follows, early endosomes fuse to form late endosomes resulting in the invagination of the endosomal membrane into the lumen and the formation of intraluminal vesicles (ILVs) in MVEs. Ceramide triggers the budding of exosome vesicles into MVEs [23]. After vesicle accumulation, the MVEs have several fates; (i) be directed to the lysosome for degradation (e.g., EGF), (ii) be recycled to the TGN, or (iii) be fused with the plasma membrane resulting in the release of the ILVs known as exosome secretion [24].

ILV biogenesis and secretion are mainly driven by the endosomal sorting complex required for transport (ESCRT) machinery, which enables vesicle budding and cargo sorting in MVEs. ESCRT machinery comprises five core ESCRT complexes, ESCRT-0, -I, -II, -III, and Vps4 [25]. The key subunit ESCRT-0 hepatocyte growth factor-regulated tyrosine kinase substrate (Hrs) recognizes and sorts ubiquitinated cargoes to phosphatidylinositol-3-phosphate (PI3P) enriched endosomal compartments [25,26]. PI3P is a phospholipid mostly found in early and late endosomes and promotes cargo organization through Hrs interaction [27]. Subsequently, ESCRT-0 recruits ESCRT-I and interacts with the ESCRT-subunit tumor susceptibility gene 101 (Tsg101). ESCRT-I and ESCRT-II promote endosomal inward budding around ubiquitinated protein clusters. The ESCRT-III charged multivesicular body protein-6 (CHMP6) subunit binds ESCRT-II and recruits CHMP4 to polymerize as a coil around the budding ILV pouch’s neck. Next, CHMP3 is added, then the bud cleaves, forming EVs, followed by ESCRT-III by Vps4 in an ATP-dependent manner [25,26].

#### 2.2.2. Chaperones and Kinases Promote Exosome Biogenesis and Secretion

Many studies have shown that exosomes are more secreted by cancer cells than normal cells [28]. Moreover, exosomes are more secreted in advanced cancer cells than in low-grade cancer cells [6,7,29]. Chaperones are involved in exosome biogenesis and secretion. Cdc37 is a chaperone assisting protein kinase folding, including membrane-associated tyrosine kinase Src [30]. CDC37 is essential for exosome biogenesis and secretion [3].

Protein kinases are involved in exosome biogenesis and secretion. c-Src in endosomal membranes promotes exosome secretion [4]. Thus, it is implicated that protein folding of SRC by CDC37 is crucial in exosome secretion. Ulk1 is a kinase activating autophagy (Unc-51-like autophagy activating kinase), particularly in response to amino acid withdrawal. Dozens of small molecule compounds targeting ULK1/ULK2-mediated autophagy in cancer have been tested [31]. The inhibition or activation of ULK1 is often effective in overcoming cancer drug resistance to tyrosine kinase inhibitors (TKI), selective BRAF inhibitors, 5-fluorouracil (5-FU), KRAS drugs, doxorubicin, tamoxifen, and crizotinib [31].

Lipid kinase VPS34 plays key roles in autophagy and immune evasion. ULK1 induces autophagy by phosphorylating Beclin-1 and activating VPS34 lipid kinase [5]. Inhibition of Vps34 reprograms cold into hot, inflamed tumors and improves anti-PD-1/PD-L1 immunotherapy [32].

The Cdc37-Hsp90 chaperone complex regulates Ulk1- and Atg13-mediated mitophagy, the selective degradation of mitochondria by autophagy [33]. Thus, Cdc37 regulates autophagy, mitophagy, and exosome biogenesis and secretion. Hsp90 is a major cargo of exosomes [6,29], found in exomeres [18] and non-vesicular fractions [34,35]. Hsp90-EVs and non-vesicular Hsp90 were increased upon heat stress [3] and in 3D cell culture with enhanced cancer stemness [10]. These are rationales for the term ‘stress EV’ or ‘stressome’, although further investigation is needed. High expression of CDC37 and HSP90 is a cause of malignant exosome secretion, and siRNA targeting CDC37 and HSP90 inhibited exosome secretion in carcinomas [3,6,36]. Additionally, extracellular HSPs can play immunogenic and immunosuppressive roles, depending on the immune cells and their receptors that detect HSPs [37,38].

### 2.3. Autophagic EVs: Autophagosome–Endosome Fusion to Secrete Amphisomes

Autophagy (or autophagocytosis) is the natural, conserved degradation of the cell that removes unnecessary or dysfunctional components through a lysosome-dependent regulated mechanism [39]. Autophagy begins with the sequestration of a portion of the cytoplasm by a membraneous organelle called a phagophore (Figure 1). The resulting vacuole (autophagosome) can fuse with multi-vesicular endosomes (MVE) to form an ‘amphisome’ [40,41,42]. Amphisomes contain a mixture of endosome- and autophagosome-derived molecules, such as LC-3 and CD63. Amphisomes subsequently fuse with (i) a lysosome to have its mixed autophagic/endocytic content degraded by lysosomal enzymes in ‘autolysosomes’ or (ii) plasma membrane to secrete the mixture of exosomes and autophagic contents, which is also called exophagy (Figure 1 and Table 1). Cytosolic DNA and nucleosomes (dsDNA/histones) are secreted with exosomes via the autophagy–amphisome pathway. Nucleosomes are released via active release of amphisome (LC3+) and passive release by cell death. Histones are also detected as non-vesicular (NV) proteins.

### 2.4. EMT Is Associated with EV Release and Immunosuppression

EV production often correlates with tumor cell transformation, such as epithelial-to-mesenchymal transition (EMT) [3,6,9,28,43] and cancer stemness [10,44]. Cdc37-Hsp90 chaperones promote EV release coupled with EMT in cancer [3,6,45]. Moreover, recent studies suggest that the EMT progression is correlated with higher PD-L1 expression, immunosuppression, and immune evasion by M2 macrophages, myeloid-derived suppressor cells (MDSC), and Treg cells. In contrast, the epithelial tumors with lower PD-L1 expression and less Treg and MDSC are susceptible to immune attack by M1 macrophages and killer T cells [46].

Of note, recent studies have shown that EMT is rarely executed as an on/off phenomenon in cancer, while the process is rather gradual and often remains incomplete, termed partial EMT, hybrid EMT, or hybrid E/M [47,48,49]. A hybrid EMT state provides maximal stemness, tumor initiation capacity, and the ability to adapt to environmental changes [50]. Prostate cancer spheroids vastly co-express stemness (CD44v9+ EpCAM+ CD133+ ESRP1/2+), epithelial (E-cadherin+), and mesenchymal (Vimentin+) markers, and markedly release EpCAM+ CD9+ EVs [10].

### 2.5. Matrix Vesicles

#### 2.5.1. ECM in the Tumor Microenvironment

ECM are crucial components of the tumor microenvironment. Tumor ECM are mainly composed of collagen, proteoglycan, fibronectin, elastin, hyaluronan, and laminin [51], while additional ECM molecules, including agrin and tenascin, are also detected in cancer cell-derived EVs [3]. ECM is produced mainly by CAFs and cancer cells, which provide a scaffold for immune cells, endothelial cells, CAF, cancer cells, and their communication. ECM is a storage and source for matrix vesicles and extracellular ligands (growth factors, cytokines, and chemokines), which are released upon MMP-dependent proteolysis [52] (Figure 1). ECM is often bound to integrins anchored to membranes of cells and EVs and thus mediate cell–matrix and vesicle–matrix interactions. For example, EVs bind to immune cells, such as B cells and reticulocytes, via their surface β1 integrins [53,54].

#### 2.5.2. ECM-Rich Microenvironment Is a Risk of Poor Prognosis in Cancer

ECM-rich tumors are at high risk of prognosis. ECM profiles determine the risk and prognosis of the squamous cell carcinoma (SCC) subtype of non-small cell lung carcinoma (NSCLC), defined by a multi-omics data analysis [55]. Over 80% of patients with pancreatic ductal adenocarcinoma (PDAC) are diagnosed at a late stage and are locally advanced or with concurrent metastases. The aggressive phenotype and relative chemo- and radiotherapeutic resistance of PDAC are thought to be mediated largely by its prominent stroma, which is supported by ECM [56].

#### 2.5.3. ECM Mediate Tumor Malignancy

ECM forms a barrier against anticancer drugs, killer immune cells, phagocytes, oxygen, and glucose [57]. Moreover, ECM deposition in tumors increases matrix stiffness that stimulates mechano-signaling transduction [58,59]. The ECM deposition and stiffness are often increased in obese adipose tissues within the TME [60]. MMPs in obese adipose tissues excessively generate ECM fragments and matrix vesicles, which act as signaling molecules in the TME.

#### 2.5.4. ECM–EVs Interaction

Matrix vesicles include extracellular matrix-bound vesicles and matrix-coated vesicles [61] (Figure 1 and Table 1). Matrix-bound vesicles are embedded in ECM around cells in tumors and stroma and normal connective tissues, such as bone and cartilage. Matrix-coated vesicles exist in the tumor microenvironment, bodily fluids [62], and cell culture supernatants [3,29]. CAFs and cancer cells are proposed as major producers of matrix vesicles. Matrix-coated vesicles interact with ECM at distant organs to promote niche formation and vasculature regulation [63]. Matrix-coated vesicles interact with ECM can result in the reprogramming of tumor-infiltrating immune cells, enabling immune cells to play essential roles during tumor progression, and promote niche formation and vasculature regulation.

Heparan sulfate proteoglycans coating EVs play crucial roles as biomarkers and bioactive molecules. Glypican-1 identifies cancer exosomes in blood and detects early pancreatic cancer [64]. MMP9 associated with heparan sulfate chains of GPI-anchored cell surface proteoglycans mediate the motility of murine colon adenocarcinoma cells LuM1. Therefore, it is proposed that MMPs are decorated on the EV surface matrix and play key roles in cancer progression and niche formation.

#### 2.5.5. Matrix Moonlighting Metalloproteinases (MMPs)

Matrix metalloproteinases (also known as moonlighting metalloproteinases) modulate EVs in multiple ways: (i) release of EVs from ECM, (ii) penetration of EVs into tissues and cells, (iii) dissemination of EVs to multiple organs, and (iv) development of niche (Table 2). Growing evidence indicates that cancer cell-derived EVs transfer oncogenic proteins and nucleic acids that modulate the activity of recipient cells and promote tumor initiation, invasion, and metastasis. Matrix/moonlighting metalloproteinases (MMPs), especially MMP3 and MMP9, are pro-tumorigenic cargos of EVs in cancer [65]. Notably, MMP3 in colon cancer EVs plays vital roles in tumorigenesis and metastasis [7,8,66]. MMP3 in EVs are transferred into recipient cell nuclei and transactivate pro-tumorigenic genes, such as cellular communication network factor 2 (CCN2) and HSPs [7,67,68,69].

### 2.6. EV-Mediated Molecular Transfer

EVs can transfer bioactive molecules to neighboring and distant recipient cells. EV-derived proteins are functional and/or degraded in lysosomes in recipient cells. Exosomal miRNAs can target mRNA in recipient cells. Exosomal mRNA can be translated into a bioactive protein in recipient cells. EV-mediated molecular transfer mechanisms are variously proposed, including direct membrane fusion, endocytosis, macropinocytosis, or cell type-specific phagocytosis [77,78]. Endosomal escape is required for EV molecules to be transferred into cytoplasm or nuclei in recipient cells.

#### 2.6.1. Protein S-Palmitoylation Regulates EV Protein Sorting and Molecular Transfer

Protein palmitoylation is crucial for sorting cargo proteins into EVs and transferring them from EVs into recipient cells [79,80]. S-palmitoylation is a reversible lipid post-translational modification involved in different biological processes, such as the trafficking of membrane proteins, achievement of stable protein conformations, and stabilization of protein interactions. Protein palmitoylation is the covalent attachment of fatty acids, such as palmitic acid, to cysteine, i.e., S-palmitoylation. Palmitoylation enhances the hydrophobicity of proteins and contributes to their membrane association [9]. Indeed, palmitoylation signal-fused GFP was efficiently sorted into EVs and transferred into recipient cells [18]. Alix S-palmitoylation influences its interaction with CD9 [81]. Common palmitoylated proteins in different cancer types are EGFR, RAS (KRAS4A, NRAS), CD82 tetraspanin, CD44, PD-L1, and CKAP4. Additional palmitoylated proteins in cancer are Integrins, WNTs (Wnt1, 2B), cell adhesion molecules (EpCAM, MCAM), claudin 3, caveolin-1, Rab7a, estrogen receptors (ER α and β), Fas, IFNGR1, FLT3-ITD, LAT2, YKT6, VAMP3 [82], GP130, Smad3, and GLUT1 [83].

#### 2.6.2. Transfer of Oncogenic Factors

EVs could cause cellular reprogramming and genetic alterations by transferring their cargo contents, such as oncoproteins, lipids, mRNAs, and noncoding RNAs, defined as ‘oncosomes’. Increasing evidence elucidated that tumor cells release EVs to reprogram normal and stromal cells in the tumor microenvironment to provoke tumor initiation, progression, metastasis, and drug resistance. For a great example, mutant KRAS [84] and mutant EGFR [84,85] were found in EVs and transferred to recipient cells leading to cancer progression [86,87]. Tumor oncosomal MMP3 is transferred to recipient cells and alters transcriptional programs, such as cellular communication network factor 2 (CCN2) expression, in the tumor microenvironment [7,66].

### 2.7. Markers Defining EVs and Finding Biomarkers from EVs

#### 2.7.1. Protein Markers of EVs

EVs contain a variety of molecular cargo such as proteins, long and small RNA, DNA, lipid, glycan, minerals, and metabolites [88,89,90,91,92,93,94,95,96]. EV membranes are enriched in lipids, such as cholesterol, sphingomyelin, and ceramide. The EV contents vary greatly depending on the originating cell. Classical exosome markers, such as tetraspanins (CD9, CD63, CD81, and CD82), HSPs, Rabs, and Alix, are often lost from exosomes in some pathophysiological conditions and found in other EV types, such as large EVs [29,90,96,97,98,99] (Table 1).

Tetraspanins (CD63, CD9, and CD81) were used as standard exosome markers in classical exosomes. However, EV proteome analyses have revealed that these tetraspanins are often lost from exosomes or detected at shallow levels [3,29]. Moreover, CD9-positive large exosomes (CD9-Exo-L) and CD63-positive small exosomes (CD63-Exo-S) were found in separated fractions released by macrophage-like cells differentiated from the THP-1 cell line, indicating the diversity of exosomes [18].

#### 2.7.2. Stem Cell Markers on EV Surface

Various stem cell markers are secreted with EVs. These include cancer stem cell (CSC) markers: (i) surface receptors, including EpCAM/CD326 [6,10,29], CD44 variants [100], Notch-ligand Dll4 [101], prominin/CD133 [102], (ii) extracellular ligands, including Wnt [103], and (iii) functional enzymes (ALDH and MMPs) [7,8]. These CSC factors in EVs facilitate communication between cancer cells and the TME.

Note that these CSC markers are also found in normal stem cell-derived EVs. MSC surface markers (CD44, CD73, and CD90) are found in human MSC-derived EVs [104]. Hair follicle- and adipose tissue-derived MSCs released EVs carrying MSC markers (CD44 and CD105) with EV markers (CD9, CD63, and CD81) [105]. Endothelial cells produce and release CD44-containing EVs upon TNFα stimulation [106]. Canonical exosome markers, including CD133, CD63, CD9, and HSP70, were found in all EV fractions of oral fluids [107].

#### 2.7.3. EV as a Source of Biomarkers

Bodily fluids, such as blood, saliva, cerebrospinal fluid, lymph fluid, sweat, tears, urine, milk, and seminal fluid, have been used as sources of pathophysiological biomarkers. EVs are often released and found in these bodily fluids. Therefore, researchers have investigated EVs for screening and discovering novel biomarkers of pathophysiological conditions and diseases, including cancer. These biomarkers include diagnostic biomarkers, predictive biomarkers, and pre-symptomatic disease state biomarkers.

EVs, including exosomes, often contain microRNAs (miRNA), mRNA, and long noncoding RNAs (lncRNA). Many EV RNA (or exosomal RNA) have been established as biomarkers, demonstrated to be transferred to and exert their function in recipient cells [90,95,96,108,109,110,111,112,113]. Moreover, additional types of noncoding RNAs have been identified in EVs, including circular RNA (circRNA) and PIWI-interacting RNA (piRNA).

## 3. Tumor EVs Develop the Immunosuppressive and Resistant Microenvironment

**Figure 2:**
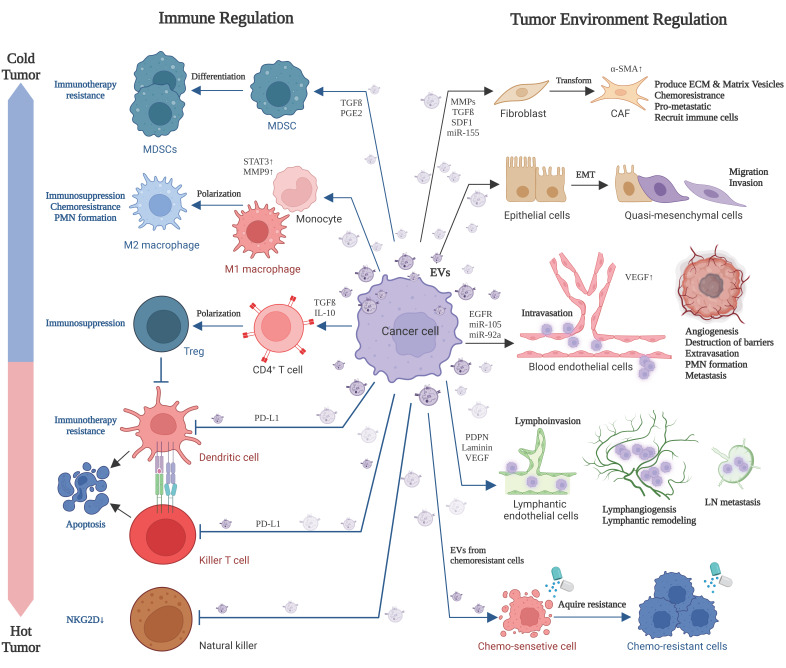
Cancer cell-derived EVs affect various cell types in the tumor microenvironment. Cancer EVs play crucial roles in immunosuppression, immune evasion, apoptosis of immune cells, and immunotherapy resistance. Thus, cancer EVs change hot tumors into cold ones. Moreover, cancer EVs affect nonimmune cells, including cellular transformation (including EMT), chemoresistance, tumor matrix production, destruction of biological barriers, angiogenesis, lymphangiogenesis, and pro-/premetastatic niche (PMN) formation.

It has become clear that tumor progression is regulated not only by the autonomous growth of cancer cells but also by the microenvironment surrounding the cancer cells, called the tumor microenvironment. The tumor microenvironment comprises various cells; EVs; ECM (Table 2); extracellular ligands (growth factors, cytokines, and chemokines); and conditions. The major cell types in the TME are CAFs; tumor endothelial cells; adipocytes; and immune cells, including TAMs, MDSCs, dendritic cells, and T cells (Figure 1 and Figure 2). Noncellular key components in the TME are ECM, EVs, and extracellular ligands. Key conditions of the TME include hypoxia, acidity (pH), and cell death [9]. Cell death, damage, and stress promote EV production and release [3].

The cell stress response is an essential system endowed with every cell for responding and adapting to extracellular environmental stimulations. Tumor cells are characteristically exposed to various stresses from the microenvironment, such as immune/inflammatory stress [38], therapeutics [114], hypoxia [9,10,44], acidification [115], heat stress or hyperthermia [116,117,118], endoplasmic reticulum stress, replication stress, oxidative stress, mechanical stress, osmotic stress, genotoxic (DNA damage) [119,120], and proteotoxic stress [121,122,123].

Individual TACs dynamically interact with each other and contribute to creating a unique microenvironment for neoplastic cells. Cancer cells and TACs communicate with each other via delivering EV cargo molecules to induce phenotypic modifications, causing cancer propagation (Figure 2). Many studies have proved the involvement of cancer EVs in the modulation of TACs in the tumor microenvironment [9] (Table 3). Along with mediating cell-to-cell communication, tumor exosomes develop cancer therapy resistance [124]. Moreover, recent studies discovered that EVs derived from tumor microenvironmental cells, such as CAFs and immune cells, alter cancer cell phenotypes (Table 4).

### 3.1. Tumor–CAF Communication to Develop Chemoresistance

#### 3.1.1. CAF Differentiation

Fibroblasts are critical components of tumor stroma, while recent studies have evoked the existence of CAFs. CAFs include myofibroblasts and are differentiated from mesenchymal stem cells (MSCs). Myofibroblasts constitute a significant component of the tumor stroma and mediate angiogenesis, which can be modulated by the cancer EVs [7,155,156]. It is worth noting that tumor stroma rich in myofibroblastic cells can maintain tumor growth, vascularization, and metastasis. Exosomal TGF-β promotes the differentiation of fibroblasts into myofibroblasts through the SMAD signaling pathway [24,126]. Further, gastric cancer-derived EVs carry TGF-β that activates the Smad pathway conducive to generating functional CAFs from umbilical cord-derived MSC [157]. Breast cancer-derived EVs stimulate the differentiation of MSCs in adipose tissue into a myofibroblast-like phenotype with a significant increase in α-SMA and other pro-tumorigenic factors, such as VEGF, SDF-1, TGF-β, and CCL5 [125]. The dependency of such stromal cells highlights the involvement of Pancreatic cancer-secreted miR-155 in EVs implicated in the conversion from normal fibroblasts into CAFs [158]. Prostate cancer-derived EVs provoke the MSC differentiation into myofibroblastic cells with an increase in VEGF-A, resulting in proangiogenic functions [159]. These studies indicated that tumor EVs play critical roles in augmenting CAFs, myofibroblasts, MSCs, and TECs.

Validation at the single cell RNA sequencing-based transcriptional, and mass spectrometry-based protein levels in several experimental models of cancer and human tumors reveal spatial separation of the CAF subclasses attributable to different origins, including the peri-vascular niche, the mammary fat pad, and the transformed epithelium [160].

Moreover, MSC-derived EVs promote cancer therapy resistance by activating EMT, evading apoptosis, increasing cancer stemness and dormancy, and increasing immunotherapy resistance [149]. On the other hand, MSC-derived exosomes have been used as novel vehicles for the delivery of miRNAs in cancer therapy [150].

#### 3.1.2. CAF-Derived Exosomal miRNAs Promote Chemoresistance and Metastasis

The CAF-derived exosomal microRNA signature supports the communication between tumor cells and other stromal residents in the TME, which promotes cancer progression and therapeutic resistance [161,162,163]. In esophageal cancer, cisplatin resistance was correlated to exosomal miR-27a/b and its target TGF-β [127]. miR-522 overexpression in CAFs was associated with cisplatin/paclitaxel resistance of gastric tumor through activation of ubiquitin-specific protease 7 (USP7)/hnRNPA1 axis, inhibiting arachidonate lipoxygenase 15 (ALOX15) and ultimately decreasing chemosensitivity [143]. EV-enriched miR-196a is transferred from CAF to adjacent tumor cells inducing platinum resistance [144]. Additionally, miR-164a and SNAI1 are delivered directly from CAF to pancreatic cancer cells via EVs, leading to gemcitabine (GEM) resistance of tumor cells. The resistance can be reversed by treatment with GW4869, an inhibitor of exosome release [145,147]. miR-106b from CAFs was also transferred to cancer cells, which conferred GEM by targeting TP53INP1 (Tumor protein p53 inducible nuclear protein 1) [164]. Additionally, miR-21 was reported in GEM-induced chemoresistance [165]. Further, miR-21-rich EVs released from cancer-associated adipocytes significantly reduced tumor cells’ sensitivity to paclitaxel by targeting apoptotic protease activating factor-1 (APAF1) in the ovarian neoplasm microenvironment [166]. Additionally, EV-enriched lncRNA H19 was transferred from CAFs to adjacent colorectal cells, activating the Wnt/ß-catenin signaling pathway and inducing chemoresistance [167,168].

Exosomal miR-500a-5p derived from CAFs promotes breast cancer cell proliferation and metastasis by targeting USP28 [146] (Figure 2).

### 3.2. Angiogenesis, Extravasation, and Intravasation Induced by EVs

#### 3.2.1. Blood Endothelial Cells Support Tumor Progression and Metastasis

Tumor blood endothelial cells (TECs) lining tumor blood vessels ensure nutrients pass into tumor tissues [169]. TECs are abnormal in morphology, function, and gene expression [170]. TECs support tumor cells disseminating to the distal sites via extravasation and preserve them from anoikis, thereby promoting tumor metastasis [171]. TECs can also release angiocrine factors, such as VEGF, to support tumor progression [172,173]. Abnormal characteristics of TECs are caused by the tumor microenvironment, such as hypoxia, that promotes the production of VEGF and increases vascular permeability and genetic instability in TECs [174]. While TECs adhere to the endothelia of venules, they will enter circulation, exit the bloodstream, and position themselves upon distance endothelium surfaces and subsequent metastatic growth [175,176].

#### 3.2.2. Chemokines and Growth Factors Induce Angiogenesis

Phosphatidylserine (SP), the inner bilayer of the intact cellular membrane, and P-selectin glycoprotein ligand-1 (PSGL1) are considered to work together, promoting the EV to adhere to the endothelium [166]. The phenotypic alterations of TECs were led by EVs that contain growth factors and receptors, such as VEGF and its receptor VEGFR1 [177,178], SDF1/CXCL12 [179,180], FGF-4 [181], EGF [182], adrenomedullin [183], and TSP-1 [184]. Moreover, EGFR-positive tumor-derived EVs promote angiogenesis by reprograming TECs into VEGF-secretion phenotype [87]. CXCR4, a receptor for SDF1, is overexpressed in TECs. At the same time, a CXCR4 antagonist (plerixafor, also known as AMD3100) induced tumor angiogenic inhibition-triggered necrosis (TAITN) in oral squamous cell carcinoma (OSCC) [179]. TAITN reduced TECs that supply oxygen to tumor cells, whereby the loss of TECs induced hypoxia [179]. Thus, chemokine signaling plays a crucial role in tumor angiogenesis, a novel therapeutic target. Tumor-derived EVs are related to tumor growth and metastasis of HNSC and induce angiogenesis by reprogramming TECs [180]. Exosomal WNT4 from colorectal cancer stimulated β-catenin nuclear translocation in endothelial cells, which improved tumor growth and angiogenesis [129,185].

#### 3.2.3. Angiogenesis Promoted by Exosomal Noncoding RNAs

Cancer-secreted exosomal miR-105 directly destroys vascular endothelial barriers to promote metastasis by targeting endothelial tight junction protein ZO-1 [186]. Exosomes derived from leukemia K562 cells with enforced miR-92a expression enhance endothelial cell migration and tube formation via regulating proangiogenic protein integrin-α5 [187]. LncRNAs contained in EVs can promote tumor angiogenesis. EVs released by CD90^+^ liver cancer cells promoted angiogenesis and adhesion of endothelial cells by providing lncRNA H19 [188]. LncRNA H19 also promoted angiogenesis in glioblastoma [189]. EVs derived from lung cancer cells contained the lncRNA growth arrest-specific 5 (lncRNA GAS5), upregulating PTEN expression and inhibiting the PI3K/AKT phosphorylation, thereby increasing angiogenesis [190]. These studies indicate that tumor EVs stimulate tumor-associated BECs to promote angiogenesis.

### 3.3. Tumor-Associated Macrophages Affected by Cancer EVs

#### 3.3.1. M2 TAM Mediates Immunosuppression

Macrophages are generally divided into the pro-inflammatory M1-type and immunosuppressive M2 type. M1-polarized macrophages possess antitumor activity, whereas M2-polarized macrophages promote tumor growth [191]. Tumor-associated macrophages (TAMs) are often M2-like phenotypes. They are considered vital participants in cancer progression via the production of numerous growth factors, cytokines, and ECM remodeling molecules for stimulating cancer growth, migration, and angiogenesis [192]. Indeed, tongue cancer EVs alter the macrophage polarity into the M2-type, while HSP90 partially mediates the TAM polarization in HNSC [6]. Breast cancer-derived exosomal glycoprotein 130 (gp130) activates the IL-6/STAT3 pathway in macrophages [193], consequently increasing the macrophage survival and inducing the expression of several genes associated with tumorigeneses, such as IL-10, CXCR4, and CCL2 [131,193]. Each cytokine has a specific role in regulating tumor immune surveillance. IL-10 induces immunosuppressive effects by modulating dendritic cells and cytotoxic T cells [194], while CXCR4 is associated with proangiogenic and immunosuppressive phenotypes [131]. IL-6 and CCL2 (also known as MCP-1: monocyte chemoattractant protein 1) are associated with TAM polarization [195]. These immunosuppressive effects are inhibited by adding a GP130 inhibitor to the cancer-derived EVs [191].

CD73 (also known as 5NT: 5’-nucleotidase) in small EVs derived from HNSCC defines tumor-associated immunosuppression mediated by macrophages in the microenvironment [196]. The CD73+ sEVs phagocytosed by TAMs in the TME-induced immunosuppression. Higher CD73-high TAMs infiltration levels in the HNSCC microenvironment were correlated with poorer prognoses, while CD73+ sEVs activated the NF-κB pathway in TAMs, thereby inhibiting immune function by increasing cytokines secretion such as IL-6, IL-10, TNF-α, and TGF-β1. The absence of CD73+ sEVs enhanced the sensitivity of anti-PD-1 therapy through reversed immunosuppression. Moreover, circulating CD73+ sEVsCD73 increased the risk of lymph node metastasis and worse prognosis. This study suggests CD73+ sEVs derived from tumor cells contribute to immunosuppression and are a potential predictor of anti-PD-1 responses for immune checkpoint therapy in HNSCC, defining hot vs. cold tumors and responders vs. non-responders.

#### 3.3.2. M2 TAM-Derived EVs Induce Chemoresistance

Macrophage-derived exosomal miR-21 enhanced the PI3K/Akt signaling pathway, inhibited apoptosis by downregulating PTEN, and induced resistance to cisplatin in gastric cancer cells [151]. Likewise, miR-365 transferred by M2 macrophage-derived EVs increased the tri-phospho-nucleotide pool in pancreatic cancer cells and activated cytidine deaminase, which eventually conferred GEM resistance and supported tumor cells proliferation [152] (Figure 2).

#### 3.3.3. Potential Exosomal Oncolipid

Redundant lipids are released from cells through the release of EVs and cholesterol efflux pump proteins. Such a pump overexpressed in metastatic cancer cells was adenosine triphosphate (ATP)-binding cassette G1 (ABCG1), which co-overexpressed with ABCG2, a drug efflux pump found in CSCs [44]. The targeted silencing of ABCG1 led to EV lipid accumulation and triggered tumor cell death. These facts suggest that cancer cells can often release redundant toxic lipids. In contrast, loss of the ABCG1 pump could trigger the accumulation of redundant harmful lipids, leading to tumor cell death. Macrophages play critical roles in cholesterol transport from peripheral blood vessels to the liver. Therefore, TAMs may play vital roles in metabolizing redundant and toxic lipids released by tumor cells.

### 3.4. T Cells Affected by Cancer EVs

#### 3.4.1. Tumor-Infiltrating Lymphocytes (TIL)

It has been suggested that tumor-infiltrating lymphocytes (TILs) include tumor-reactive lymphocytes and tumor antigen-specific lymphocytes. A therapy in which tumor-reactive T cells in TILs are expanded, cultured, and infused is being attempted. At the same time, it is suggested that many cells negatively regulate the antitumor immune response, such as T reg cells, in TILs. Killer T cells, also called cytotoxic T lymphocytes (CTLs), are a group of CD3^+^ CD8^+^ T cells that exhibit cytotoxicity specifically to cells presenting antigen peptides on MHC class I molecules on the target cell surface. Killer T cells recognize antigen peptides and secrete cytotoxic granules that contain perforin and granzymes. Perforin polymerizes on the target cell membrane to form pores. Granzymes, which belong to serine proteases, invade the target cells through the pores and induce apoptosis of the target cells. Activated killer T cells are considered the master regulator of the antitumor immune response. A growing body of studies has reported the significance of CD4^+^ helper T cells in the generation and maintenance of effective cytotoxic and memory CD8^+^ T cells, known as CD4^+^ T-cell help. This phenomenon optimizes the expansion, trafficking, and effector function of CD8^+^ T cells, thereby potentiating immune-mediated tumor destruction [197,198,199].

#### 3.4.2. Apoptosis of Killer T Cells Induced by Cancer EVs

Cancer cell-derived EVs suppress these T cells, which are more sensitive to the suppressive effects of tumor EVs than other immune cells. These immunosuppressive effects of cancer EVs involve the induction of apoptosis, inhibition of proliferation and differentiation, and dysfunctionality of T cells. EVs from ovalbumin peptide (OVA)-expressing melanoma suppressed OVA-specific immune response [200]. Several studies showed tumor EVs induce T cell apoptosis through FasL, TNF, and galectin-9, located on the EV surface [137,201,202]. Furthermore, PTEN of tumor EVs appeared to regulate the PI3K/AKT pathway, leading to AKT dephosphorylation and increasing the expression of pro-apoptotic BAX and decreasing antiapoptotic Bcl-2, Bcl-xL, and MCL-1 (myeloid leukemia cell differentiation protein) in activated killer T cells [203,204,205]. Additionally, the administration of GL26 glioblastoma EVs to mice was associated with a reduction in the number of killer T cells and a decline in the IFN-γ and granzyme expression [206]. Extracellular ectonucleotidases CD39 and CD73 contribute to rising adenosine levels in the tumor microenvironment by dephosphorylating exogenous ATP and 5′AMP to form adenosine and hence attenuating the T-cell function [207].

#### 3.4.3. Treg Cells Induced by Cancer EVs

Regulatory T cells (Tregs) are an immunosuppressive subset of CD4^+^ T cells and negatively impact the immune response. TGF-β1 and IL-10 in EVs stimulate the differentiation of CD4^+^ CD25^−^ T cells into Tregs and foster the Tregs proliferation by increasing the phosphorylated SMAD2/3 and STAT3 [138]. These studies demonstrated that cancer EVs suppress killer T cells through activating pro-apoptotic signals and promoting differentiation of T cells into Tregs, immunosuppressive T cells (Figure 2).

### 3.5. MDSCs Potentiated by Cancer EVs

#### 3.5.1. MDSC—A Master Regulator of Immunosuppression

Myeloid-derived suppressor cells (MDSCs) are a heterogeneous population of immature myeloid cells unable to differentiate into DCs, macrophages, or granulocytes. MDSCs are one of the main drivers of immunosuppression in the tumor microenvironment. MDSCs exhibit a robust suppressive capacity against T cells and NK cells antitumor activity, recruiting immunosuppressive Tregs and creating a microenvironment favorable for immunosuppression and tumor progression. Therefore, an increased MDSC frequency and activity were positively correlated with tumor progression and recurrence and negatively associated with immunotherapy efficacy and clinical outcomes [208].

#### 3.5.2. MDSC Differentiation and Recruitment Promoted by Tumor EVs

Tumor EVs promote MDSC differentiation through TGF-β and prostaglandin E2 (PGE2) in vivo. Tumor EVs also induce the expression of Cox2, IL-6, VEGF, and arginase-1 in the accumulating MDSCs. Blocking the tumor exosomal PGE2 and TGF-β activities disrupted the stimulatory effect of these EVs on MDSC and attenuated MDSC-mediated immunosuppression [139]. It was recently shown that a chemokine CCL bound to cancer EVs determines uptake by CXCR-expressing cells [76]. Resident stroma-secreted chemokine CCL2 recruits MDSCs in the tumor microenvironment [140].

These findings suggested that tumor EVs potentiate the immunosuppressive roles of MDSCs in regulating NK cells and T cells. Therefore, a potential therapeutic strategy is blocking immunosuppressive cytokines on the tumor EVs to attenuate the unfavorable immunosuppression by MDSCs.

### 3.6. Tumor EVs Downregulate a Killing Factor of NK Cells

#### 3.6.1. NK Cells Express a Killing Factor NKG2D

Natural Killer (NK) cells have abilities to kill tumor cells and virus-infected cells without prior sensitization. NK group 2 member D (NKG2D) protein is a type-II transmembrane receptor expressed on the surface of NK cells and killer T cells. In NK cells, NKG2D mediates the direct killing of target cells. In CD8^+^ killer T cells, it acts as a costimulatory receptor leading to the activation of the T-cell receptor (TCR) and T-cell effector function [141,142].

#### 3.6.2. Tumor EVs Downregulate NKG2D Expression on NK Cells

Human prostate cancer-derived EVs express ligands for NKG2D on their surface, which selectively decreases the expression of the receptor NKG2D on NK and CD8^+^ killer T cells in a dose-dependent manner, leading to impairing the cytotoxic function of these killer cells and promoting tumor immune escape [141]. Moreover, human prostate cancer cell EVs (derived from PC-3 and DU-145 cell lines) express NKG2D ligands on their surface that downregulated NKG2D expression in effector lymphocytes [209]. Exosomal TGF-β might be involved in NKG2D downregulation, because cell activity and NKG2D expression were restored by using TGF-β-neutralizing antibody [210]. These studies indicated that NKG2D on the surface of NK cells is crucial for killing tumor cells. In contrast, tumor EVs often express NKG2D ligand that downregulates NKG2D on NK cells.

### 3.7. Tumor EVs Induce the Immune Checkpoint of DCs

Dendritic cells (DCs) are professional antigen-presenting cells (APCs) derived from bone marrow and play a central role in initiating the immune response. DCs undergo maturation and travel to lymph nodes after capturing the antigens with their recognition receptors. DCs present the captured antigens to naïve T cells for their activation and polarization, establishing links between innate and adaptive responses [211,212,213].

Tumor EVs block DCs maturation to decrease the T-cell immune response [134]. In this study, EVs from Lewis lung carcinoma (LLC) or 4T1 breast cancer cells blocked the differentiation of myeloid precursor cells into CD11c+ DCs and induced cell apoptosis. Tumor EV treatment inhibited the maturation and migration of DCs and promoted the immune suppression of DCs. The treatment of tumor EVs drastically decreased CD4+ IFN-γ+ Th1 differentiation but increased the rates of Treg cells. The immunosuppressive ability of tumor EV-treated DCs was partially restored with PD-L1 blockage. These data suggested that PD-L1 played a role in tumor EV-induced DC-associated immune suppression.

### 3.8. Tumor EVs Regulate Lymphatic Endothelial Cells (LECs) and Lymph Node Metastasis

#### 3.8.1. Tumor Lymphangiogenesis and Lymphoinvasion

Regional lymph nodes (LNs) are the primary sites of lymphatic drainage from organs, and the extent of their involvement in cancer is a strong predictor of disease relapse and patient survival. Lymph node metastasis (LNM) of tumors is an established indicator of poor prognosis in patients. The migration of cancer cells into the lymphatic circulation when entering the LNs is greatly facilitated by tumor lymphangiogenesis, a process that generates new lymphatic vessels (LVs) from preexisting conduits [214,215]. Clinical studies have shown that the production of lymphangiogenic factors and the occurrence of lymphangiogenesis correlate with disease outcomes in various tumor types. Several soluble factors produced by lymphatic endothelial cells (LECs), cancer cells, and neighboring stromal cells have been implicated in the regulation of lymphangiogenesis and lymphoinvasion (including VEGFA, VEGFC, VEGFD, PDGFBB, and angiopoietin [215,216]. In addition, chemokine signaling pathways (CCL21/CCR7 and CCL19/CCR7) mediate the homing of immune cells to LNs [217]. Other chemokine receptors, such as CXCR2, CXCR3, and CXCR4, also contribute to lymphangiogenesis [218,219,220].

#### 3.8.2. Podoplanin (PDPN) Regulates Tumor Lymphangiogenesis and Lymphoinvasion

Podoplanin (PDPN) is a heavily O-glycosylated small mucin-type transmembrane glycoprotein with a wide variety of functions, including the regulation of cell motility and adhesion [221]. The upregulation of PDPN correlates with malignant progression in several tumor types [222]. The roles of cancer cell-derived PDPN in tumor progression have been well documented [223,224]. PDPN is a component of tumor EVs that reprograms EV biogenesis and release and modulates lymphatic vessel formation [225].

Moreover, PDPN is strongly expressed by LECs and widely present in tumor-associated cells, e.g., in TAMs. Podoplanin-expressing macrophages (PoEM) promote lymphangiogenesis and lymphoinvasion in breast cancer [226]. This study showed that a triad interaction of PoEM, GAL8-expressing lymphatic vessels, and invading breast cancer cells promotes metastasis. PoEM remodels the ECM, stimulating lymphangiogenesis and releasing previously trapped cancer-promoting factors that facilitate cancer cell lymphoinvasion. The MMP-dependent matrix remodeling and growth factor release are essential for PoEM-mediated lymphangiogenesis and lymphoinvasion.

#### 3.8.3. Tumor EVs Regulate Lymphangiogenesis

LECs regulate tumor lymphangiogenesis through the uptake of EVs packed with different biologically active contents [136]. Laminin 332 (or laminin γ2) was significantly upregulated in exocrine bodies isolated from OSCC patients with positive LNM compared to healthy people and patients without LNM [227]. The uptake of EVs by LECs was dependent on integrin. PDPN is a component of EVs that reprograms EV biogenesis and release and modulates lymphatic vessel formation [225]. Moreover, EV can promote tumor lymphangiogenesis by intracellularly transferring lymphangiogenic factors VEGFC and CXCR4 [228].

Several lncRNAs and miRNAs regulate lymphangiogenesis and LNM in various cancer types. These lncRNAs include BLACAT2 [229], ANRIL [230], and LNMAT2 [231]. Exosomal miRNAs that regulate lymphangiogenesia dn LNM are miR-296, miR-296, miR-142-5p, miR-221-3p, miR-1468-5p, and miR-132 (reviewed in [136]).

#### 3.8.4. Tumor EVs Promote LN Metastasis

EVs are believed to reach LNs before tumor cells and form a premetastatic niche. Tumor-draining LNs undergo massive remodeling, including expansion of the lymphatic sinuses, a process that has been linked to lymphatic metastasis by the creation of a premetastatic niche. It was shown that premetastatic niche formation by EVs is CD44v6 dependent [232]. In this study, neither CD44v nor EVs alone suffices for (pre)metastatic niche formation. Instead, CD44v suffices for assembling a soluble matrix, which allows EVs, independent of their origin from poorly or highly metastatic cells, to modulate (pre) metastatic organ cells for tumor cell embedding and growth.

Of note, melanoma-derived EVs mediate lymphatic remodeling and impair tumor immunity in draining lymph nodes [135]. In this study, EVs derived from melanoma cells are rapidly transported by lymphatic vessels to draining LNs, where they selectively interact with LECs and medullary sinus macrophages. The uptake of melanoma EVs by LN-resident LECs was partly dependent on lymphatic VCAM-1 expression. Melanoma EVs shuttled tumor antigens to LN LECs for cross-presentation on MHC-I, resulting in apoptosis induction in antigen-specific CD8+ T cells.

#### 3.8.5. Exosomal Biomarkers of Lymph Node Metastasis (LNM)

PD-L1 levels on EVs, but not levels of soluble PD-L1, associated with LNM in head and neck squamous cell carcinoma (HNSCC) patients. In patients with OSCC, the levels of laminin-332 in plasma EVs in LNM+ patients were significantly higher than those in LNM- patients, indicating that laminin-332 carried by EVs could be used to detect OSCC LNM [227]. Moreover, LNM markers were identified from preliminary EVs profiling in post-operative drainage fluid (PDF) after neck dissection in OSCC [233]. PDF-EVs were mainly derived from epithelial cells and immune cells, including EGFR+ epithelial cells, CD19+ B cells, CD41a (integrin α2B)+ platelets, CD56+ NK cells, CD235+ red blood cells, CD8+ killer T cells, and CD144 (VE-cadherin)+ vascular endothelial cells. Many (2134) proteins in the PDF-EVs were identified, and 313 were differentially expressed between the LNM+ and LNM- groups. Metabolic proteins, such as EHD2 and CAVIN1, were expressed at higher levels in the LN+ group than in the LN- group, and EHD2 and CAVIN1 in the PDF were positively correlated with lymph node metastasis.

Noncoding RNAs are found to be biomarkers of LNM, including human circRNA 0056616 in lung adenocarcinoma [234], miR-146b-5p and miR-222-3p in thyroid cancer [235], and miR-21 in HNSCC [236].

## 4. EVs Contribute to Immunosuppression and Chemoresistance

### 4.1. Hot Tumors and Cold Tumors

Recent clinical studies have revealed responders and non-responders to anticancer drugs, including immunotherapy and chemotherapy. Additionally, tumor immunology has defined ‘hot tumor’ and ‘cold tumor’. Hot tumors are inflamed tumors that often respond to immune checkpoint inhibitors (ICI). CD8+ T cells infiltrate, but their effects are inhibited in hot tumors. ICI can activate tumor immunity in hot tumors. Cold tumors are noninflamed tumors that excluded or deserted T cells [237]. CD8+ T cells are absent from deserted tumors and their periphery. CD8+ cells accumulate but do not efficiently infiltrate T-cell-excluded tumors [237].

### 4.2. Exosomal PD-L1 Contributes to Immunosuppression

Exosomal PD-L1 contributes to immunosuppression and is associated with the anti-PD-1 response [238]. Tumor cells release PD-L1 with EVs, which release, cancel, and neutralize ICI and PD1+ T cells [45]. Exosomal PD-L1 has been shown as a mechanism underlying low-clinical responses to PD-1/PD-L1 blocking antibodies in immunotherapy [239]. The evolution of circulating exosomal-PD-L1 was tracked to monitor melanoma patients [240]. Thus, tumor exosomal PD-L1 is involved in cold tumor formation.

### 4.3. Exosomal EGFR Contributes to Immune Evasion

Tumor EGFR is also released with EVs in response to anti-EGFR antibody (cetuximab) treatment [43,45]. Similar to cetuximab, monoclonal antibody therapies induce antibody-dependent cellular cytotoxicity (ADCC) by NK cells and antibody-dependent cellular phagocytosis (ADCP) by macrophages in the innate immune system [45]. Thus, exosomal EGFR contributes to immune evasion from monoclonal antibody therapies and the innate immune system.

### 4.4. Tumor Microenvironmental EVs Contribute to Chemoresistance

Moreover, EVs mediate chemoresistance in cancer. Platinum drugs, such as cisplatin, are released with EVs from tumor cells [45]. A common mechanism of EV-mediated chemoresistance is: (i) EVs are released from cancer cells, TAMs, or CAFs in response to chemotherapies, and (ii) these EVs deliver specific cargoes into cancer cells to induce chemoresistance. These cargoes include RNAs (microRNAs, circular RNA (circRNA), PIWI-interacting RNA (piRNA), and lncRNA) and proteins.

Indeed, the transfer of LncRNA CRNDE in TAM-derived exosomes is linked with cisplatin resistance in gastric cancer [241]. Senescent neutrophils-derived exosomal piRNA-17560 promotes chemoresistance and EMT of breast cancer via FTO (fat mass and obesity-associated protein)-mediated m6A demethylation [242]. CAF-derived exosomal miR-20a suppresses the PTEN/PI3K-AKT pathway to promote the progression and chemoresistance of NSCLC [243]. Exosomes mediate autocrine and paracrine actions of plasma gelsolin in ovarian cancer chemoresistance [244]. EMT-induced exosomal miR-21 suppresses NLRP3 inflammasome activity in TAMs to enhance cisplatin resistance [132].

Moreover, the abnormal expression of ABC transporters is regulated by intracellular or exosomal noncoding RNAs through diverse mechanisms [245]. APE1 interacts with the nuclear exosome complex protein MTR4 and is involved in cisplatin- and 5-fluorouracil-induced RNA damage response [246].

Thus, EV release and molecular transfer mediate immune evasion and cancer drug resistance in non-responding cold tumors.

## 5. Conclusions

Although the term ‘exosome’ is carelessly used, many studies did not confirm exocytosis. Therefore, using the term ‘EVs’ or ’sEV’ is recommended by EV experts. Key molecules that regulate exosome biogenesis and secretion are kinases (including autophagy-activating kinase ULK1, lipid kinase VPS34, and membrane-bound kinase SRC) and a kinase-specific chaperone CDC37. EMT and cancer stemness is involved in EV biogenesis and release. Autophagy is largely involved in EV biogenesis and release. Autophagosome can fuse with endosomes to generate amphisomes, which are then secreted as autophagic EVs. Matrix vesicles and ECM are key components interacting with each other in the TME. Moonlighting MMPs are involved in the generation and function of matrix vesicles. Autophagic EVs, stressed EVs, and matrix vesicles have been discovered, but more studies are required.

Protein S-palmitoylation enables the proteins to associate with lipid membranes in cells and EVs, thus contributing to sorting the proteins to EVs and delivering them to recipient cells. Many protein markers and stem cell markers have been established for defining EVs. Nevertheless, numerous new biomarkers of diseases and pathophysiological conditions are currently being developed from EVs. Noncoding RNAs, including lncRNA, miRNA, circRNA, and piRNA, are found as biomarkers and functional RNA for establishing chemoresistance.

In the TME, cancer cell-derived EVs: (i) promote tumor angiogenesis, extravasation, and intravasation of cells and EVs; (ii) promote the differentiation of functions of immunosuppressive cells, such as MDSC and Treg cells; and (iii) change the differentiation or polarity of various cell types into pro-tumorigenic, immunosuppressive, anti-inflammatory, and chemoresistant phenotypes. These include the differentiation/polarization of fibroblasts into CAF, monocytes and TAM into M2-polarized TAM, and neutrophils into N2-polarized neutrophils; (iv) induce apoptosis in DCs and killer T cells; and (v) disable NK cells. Thus, tumor EVs are essential for establishing ‘cold’ tumors that are not or less responsive to immunotherapy.

Furthermore, tumor microenvironmental cells, such as CAF, MSC, TAM, and N2 cells, produce EVs to deliver bioactive molecules to cancer cells for inducing chemoresistance, immunotherapy resistance, dormancy, stemness, and EMT. Thus, these TACs and cancer cells mutually communicate via EVs to establish an immunosuppressive and resistant microenvironment.

## Figures and Tables

**Table 1:** Classification of extracellular vesicles.

Category	Name	EV Class	Size	Markers	Biogenesis
Exosome	Classical exosome	Small EV ^a^	40–150 nm	CD63, CD9, CD81	Multivesicular endosome
	Non-classical exosome	Small EV	40–150 nm	CD63/CD9/CD81-negative	Multivesicular endosome
Microvesicle	Classical microvesicle	Large EV ^b^	~150–1000 nm	Annexin A1, ARF6	Plasma membrane shedding
	Large oncosome	Large EV	1–10 µm	Annexin A1, ARF6	Plasma membrane shedding
	ARMM	Small EV	~40–100 nm	ARRDC1, TSG101	Plasma membrane shedding
Apoptotic EV	Apoptotic body	Large EV	1–5 µm	Annexin V, PS	Apoptosis
	Apoptotic vesicle	Small to Large EV	~100–1000 nm	Annexin V, PS	Apoptosis
Autophagic EV	Autophagic EV	Small to Large EV	40–1000 nm?	LC3B-PE, p62 dsDNA/Histones	Autophagosome-endosome fusion (Amphisome)
Stressed EV (Stressome)	Stressed EV Damaged EV	Small to Large EV	40–1000 nm?	HSP90, HSPs	Plasma membrane shedding, autophagy
Matrix vesicles	Matrix vesicles	Small to Large EV	40–1000 nm?	Fibronectin, Proteoglycans	Matrix binding and release
Exomere	Nano-particle	Non-EV	~35–50 nm	HSP90, HSPs	Stress?
Non-vesicular particles	Nano-particle	Non-EV	? (vaults: ~70 nm)	Fibronectin, dsDNA/Histones, MVP, HSPs	Unknown, Cell death

ARF6, ADP-ribosylation factor 6; ARMM, arrestin-domain-containing protein 1 (ARRDC1)-mediated microvesicle; ECM, extracellular matrix; EV, extracellular vesicle; LC3B-PE, microtubule-associated protein light chain B—phosphatidylethanolamine; MVP, major vault protein; PS, phosphatidylserine exposure; ^a^ Small EVs are <200 nm in diameter. ^b^ Large EVs are >200 nm in diameter. Note that the categories are not entirely mutually exclusive.

**Table 2:** Extracellular matrix–vesicle interaction.

Scene	Events	Ref.
1. Around producer cells	a. CAFs and cancer cells are major producers of matrix proteins.	[51,52]
	b. EVs are embedded within ECM and accumulated around producer cells.	[70]
	c. EVs and ECM mutually promote their accumulation around cells.	[70]
	d. sEVs act similar to car wheels to help cells migrate on rails of fibronectin.	[71]
	e. MMPs cleave matrix proteins to release matrix vesicles, growth factors, and chemokines.	[72]
	f. MMPs destroy ECM to increase the accessibility of proteins, EVs, and drugs to target cells.	[70]
2. In bodily fluids (or tissue culture supernatant)	a. EVs are often coated with matrix (fibronectin, proteoglycan, agrin, tenascin, hyaluronan).	[64,73]
	b. EV surface MMPs promote the dissemination of EVs.	[7]
3. At niches (at local and distant tissues)	a. EV surface integrins bind to ECM, leading to niche formation.	[74]
	b. EV surface matrices bind to ECM on the surface of recipient cells.	[75]
	c. EV surface growth factors, cytokines and chemokines determine uptake and bio distribution.	[76]
	d. MMPs loaded in EVs are transferred into recipient cell nuclei and transactivate the *CCN2* gene, encoding a matricellular protein.	[7]
	e. EV surface MMPs promote the penetration of EVs into target tissues.	[8]

**Table 3:** Influences of cancer cell-derived EVs on recipient cells.

Recipient Cells	Influences	Model	Refs.
MSCs	↑ Differentiation to proangiogenic myofibroblasts ↑ Differentiation to pro-invasive myofibroblasts	In vitro	[125,126]
Fibroblasts (CAF)	↑ Fibroblast differentiation into CAFs ↑ Create premetastatic niche	In vitro In vivo	[127,128]
Epithelial cells	↑ Initiate carcinogenic EMT	In vitro	[6,28]
Blood endothelial cells (BEC)	↑ Reprogram normal endothelial cells to TECs ↑ Promote tumor angiogenesis ↑ Destruct endothelial barrier ↑ Extravasation of tumor cells and EVs ↑ Intravasation and metastasis of tumor cells and EVs ↑ Promote premetastatic niche formation	In vivo, In vitro	[129,130]
Monocytes Macrophages (TAM)	↑ Induce immunosuppressive M2 polarization ↑ Expression of IL-10, CXCR4, and CCL2 ↑ Induce chemoresistance ↑ Initiate premetastatic niche formation ↓ Suppress NLRP3 inflammasome activity	In vitro, Ex vivo	[131,132]
Neutrophils (TAN)	↑ Induce N2 polarization ↑ Promote cancer cell migration	In vitro, In vivo	[133]
Dendritic cells (DC)	↓ Block myeloid precursor cells differentiation to DCs ↓ Induce DC apoptosis ↓ Decrease CD4+ IFN-γ+ Th1 differentiation ↑ Increase the rate of Treg	In vitro	[134]
Lymphantic endothelial cells (LEC)	↑ Lymphatic remodeling ↑ Lymphangiogenesis ↑ Immunosuppression ↑ Premetastatic niche formation ↑ Lymph node metastasis	In vitro In vivo	[135,136]
Killer T cells	↓ Inhibit proliferation and differentiation ↓ Induce apoptosis	Patient samples In vitro	[137]
Treg cells (Immunosuppressive)	↑ Promote the differentiation and proliferation	In vitro	[138]
MDSCs (Immunosuppressive)	↑ Promote MDSC differentiation ↑ Expression of Cox2, IL-6, VEGF, and arginase-1 ↓ Decrease antitumor immunotherapy efficacy	In vivo	[139,140]
Natural Killer (NK) cells	↓ Downregulate NKG2D expression	In vitro	[141,142]

CAF, cancer-associated fibroblast; CTL, cytotoxic T lymphocytes; EMT, epithelial-to-mesenchymal transition; MDSC, myeloid-derived suppressor cells; MSC, mesenchymal stem cell; TAM, tumor-associated macrophage; TAN, tumor-associated neutrophils; TEC, tumor vascular endothelial cells.

**Table 4:** Influences of cancer-associated-cells-derived EVs on cancer cells and immune cells.

Donor EVs	Recipient Cells	Functions	Model	Ref.
CAF-EVs	Cancer cells	↑ Induce chemoresistance ↑ Increase survival and proliferation ↑ Activate EMT ↑ Promote metastasis ↓ Suppress cell death (ferroptosis)	In vitro	[143,144,145,146,147]
	Immune cells	Additionally called metastasis-associated fibroblasts (MAF) ↑ Upregulation of IL-33 instigating type 2 immunity ↑ Recruitment of eosinophils, neutrophils, and inflammatory monocytes to lung metastasis	In vivo	[148]
MSC-EVs	Cancer cells	↑ Activate EMT ↑ Evade apoptosis ↑ Increase cancer stemness and dormancy	In vitro	[149]
	Immune cells	↑ Increase immunotherapy resistance	In vitro	[149]
	Cancer cells	↓ Vehicles for delivery in cancer therapy	In vitro	[150]
TAM-EVs	Cancer cells	↑ M2 TAM-EVs induce chemoresistance ↓ M2 TAM-EVs Inhibit immune surveillance ↓ Reduce cancer cells viability	In vitro	[151,152]
TAN-EVs	Cancer cells	↑ Induce chemoresistance ↑ Activate EMT	In vitro, In vivo	[153]
(Engineered) Immunocyte-EVs	Cancer cells	Chemoimmunotherapeutic nanocarrier ↓ Reduce cancer cells viability	In vitro In vivo	[153,154]

## Data Availability

Not applicable.
